# Supplementary material for: EventEpi—A natural language processing framework for event-based surveillance
Source: PLoS Comput Biol. 2020 Nov 20;16(11):e1008277. doi: 10.1371/journal.pcbi.1008277 (PMC7717563; doi:10.1371/journal.pcbi.1008277)
Supplement: S1 Text — (PDF) [file pcbi.1008277.s001.pdf]

1 Supporting information: *EventEpi* – A natural language processing framework for event-based surveillance

IDB preprocessing. The IDB was not designed to be used with machine learning algorithms. It thus contained some inconsistencies that might not disturb human users but had to be resolved before machine learning algorithms were applied. This means that a column that would ask for a case count could also contain strings (of alphabetic letters) and a column that required a disease name could contain numbers. Other problems were that the entries in the IDB were written in German but the output of EpiTator is in English. Moreover, many entries were subject to typographic errors, formatting errors, abbreviations, and inconsistent naming schemes. Rarely, the IDB contained symptoms and syndromes instead of a disease name. We discarded symptoms ("cough" and "fever") and kept syndromes when EpiTator could recognize them.

To translate German IDB entries into English, we used the MyMemory translation API [1]. Wrongly translated terms, abbreviations, or words not recognized were corrected by hand. We used handwritten rules (e.g., removal of trailing whitespace) to correct for formatting errors. To maximize the number of IDB entries that were usable by machine learning, we transformed the dataset into a tidy format [2].

Building the dataset INIG uses a fixed set of sources and evaluates all articles from those sources. A non-exhaustive list of these mandatory web pages includes several reports from WHO and its regional offices, from the European Centre for Disease Prevention and Control, ProMED Mail, Center for Infectious Disease Reseach and Policy, and outbreaknewstoday.com. By scraping websites from INIG's set of sources we could find all these articles, the *relevant* ones having the corresponding URL entered in the IDB, the rest being labeled as *irrelevant*. However it would have been too extensive to scrape all sources used in the IDB, as custom scrapers have to be built for each individual source. Therefore, we reduced the number of sources by firstly considering only sources that mostly contain epidemiological news (e.g., some websites contained mixed content of which only a fraction are outbreak news). Secondly, we chose sources that could be easily scraped. Thirdly, we picked those most frequently referenced in the IDB. Two sources stood out as being relevant, easy to scrape, and frequently entered in the IDB: World Health Organization Disease Outbreak News (WHO DON) and ProMED Mail.

The dataset was split using sklearn and numpy [3] which supported different stratification, seed, and splitting settings. We chose the least complex splitting settings which yielded slightly different

dataset sizes for the CNN (split with numpy) and the other relevance scoring algorithms (split with sklearn).

Text preprocessing The text is split into words using the NLTK word tokenizer. Additionally, we lower-cased all tokens, which is standard procedure to avoid that tokens at the beginning of a sentence are treated differently. Finally, we removed punctuation and stop words to reduce noise in our training data using NLTK's stop word list for the latter. The input of the naive Bayes classifiers was additionally lemmatized ("goes" and "going" become "go") to reduce the feature space and tokens were treated as n-grams in the range of one to four. In other words, each token was one feature, two tokens next to each other were one feature and three token in succession were a feature which has the advantage to treat phrases as one feature since proper nouns can consist of several words such as "Ebola virus disease" but are typically tokenized into three token.

Computational time and resources The classifiers were trained on a computer with a Ryzen 3600 (6×3.60 GHz) and 16 GB of RAM using only one core. All the classification algorithms took less then one minute to train except for the complement and multinomial NBC for the relevance scoring task which took around five minutes. This is mostly due to the very slow lemmatization.

Training the word embeddings using the word2vec algorithm took about 60 hours on 50 cores, each 2.8 GHz, and 128 GB of RAM.

References

1. LABS T. MyMemory;. Available from: `https://mymemory.translated.net/doc/spec.php`.
2. Wickham H. Tidy Data. Journal of Statistical Software. 2014;59(10):1–23. doi:10.18637/jss.v059.i10.
3. van der Walt S, Colbert SC, Varoquaux G. The NumPy Array: A Structure for Efficient Numerical Computation. Computing in Science Engineering. 2011;13(2):22–30.
