# Supplementary material for: EventEpi—A natural language processing framework for event-based surveillance
Source: PLoS Comput Biol. 2020 Nov 20;16(11):e1008277. doi: 10.1371/journal.pcbi.1008277 (PMC7717563; doi:10.1371/journal.pcbi.1008277)
Supplement: S1 Table — This tables lists the parameters and the vectorization methods stratified for the task and used models (naive Bayes classifier (NBC), support vector machine (SVM), k-nearest neighbors (kNN), logistic regression (LR), multi layer perceptron (MLP), and convolutional neural network (CNN)). More information on the used parameters can be found at https://scikit-learn.org. (PDF) [file pcbi.1008277.s002.pdf]

task	model	vectorization	parameters
key date extraction	Bernoulli NBC	bag-of-words	Laplace smoothing with $alpha = 0.01$<br>no n-grams<br>tf-idf transformation
key date extraction	multinomial NBC	bag-of-words	Laplace smoothing with $alpha = 0.01$<br>n-gram range of 1-3<br>no tf-idf transformation
key count extraction	Bernoulli NBC	bag-of-words	Laplace smoothing with $alpha = 0.001$<br>no n-grams<br>tf-idf transformation
key count extraction	multinomial NBC	bag-of-words	Laplace smoothing with $alpha = 0.01$<br>n-gram range of 1-4<br>no tf-idf transformation
relevance scoring	SVM	document embedding	penalty parameter of $C = 1$<br>kernel function coefficient $\gamma = \frac{1}{300}$<br>radial basis function kernel
relevance scoring	kNN	document embedding	$k = 5$
relevance scoring	LR	document embedding	L2 regularization of strength $C = 1$
relevance scoring	multinomial NBC	bag-of-words	Laplace smoothing with $alpha = 0.001$<br>n-gram range of 1 to 3 and tf-idf transformation
relevance scoring	complement NBC	bag-of-words	Laplace smoothing with $alpha = 0.001$<br>no n-grams and no tf-idf transformation
relevance scoring	MLP	document embedding	100 neurons in the hidden layer<br>rectified linear unit as activation function<br>Adam optimizer with default values [1]<br>L2 penalty to avoid overfitting
relevance scoring	CNN	word embeddings	from input to output:<br>batch size of 128<br>400 words with 300-dim word embeddings per document<br>convolutional layer with 150 filters<br>kernel size of (1,2)<br>stride of 1<br>ReLu activation function<br>max pooling over all filters<br>no padding<br>dropout of 0.5<br>fully connected layer with 256 hidden units<br>with tanh activation function<br>fully connected to two output units<br>with softmax activation function<br>Adam optimizer with default values [1]<br>10 epochs

References

1. Kingma DP, Ba J. Adam: A Method for Stochastic Optimization. arXiv. 2014;.
