# Supplementary material for: EventEpi—A natural language processing framework for event-based surveillance
Source: PLoS Comput Biol. 2020 Nov 20;16(11):e1008277. doi: 10.1371/journal.pcbi.1008277 (PMC7717563; doi:10.1371/journal.pcbi.1008277)
Supplement: S2 Table — For each classifier and label, the precision (Pre.), sensitivity (Sen.), specificity (Spec.), F1, index balanced accuracy (IBA) with α = 0.1, and sample size for both classes, relevant and irrelevant articles, of the test set is given. The best values for each score are highlighted in bold. (PDF) [file pcbi.1008277.s003.pdf]

| | **Pre.** | **Sen.** | **Spec.** | **F1** | **IBA** | ***relevant* sample size** | ***irrelevant* sample size** |
|---:|---|---|---|---|---|---|---|
| **Logistic regression** | 0.00 | 0.00 | **1.00** | 0.00 | 0.00 | 38 | 771 |
| **k-nearest neighbor classifier** | 0.00 | 0.00 | 0.99 | 0.00 | 0.00 | 38 | 771 |
| **Support vector machine** | 0.00 | 0.00 | **1.00** | 0.00 | 0.00 | 38 | 771 |
| **Multilayer perceptron** | **0.17** | **0.03** | 0.99 | **0.05** | **0.02** | 38 | 771 |
