# Supplementary figures and images for: EventEpi—A natural language processing framework for event-based surveillance
Source: PLoS Comput Biol. 2020 Nov 20;16(11):e1008277. doi: 10.1371/journal.pcbi.1008277 (PMC7717563; doi:10.1371/journal.pcbi.1008277)

task = key date extraction

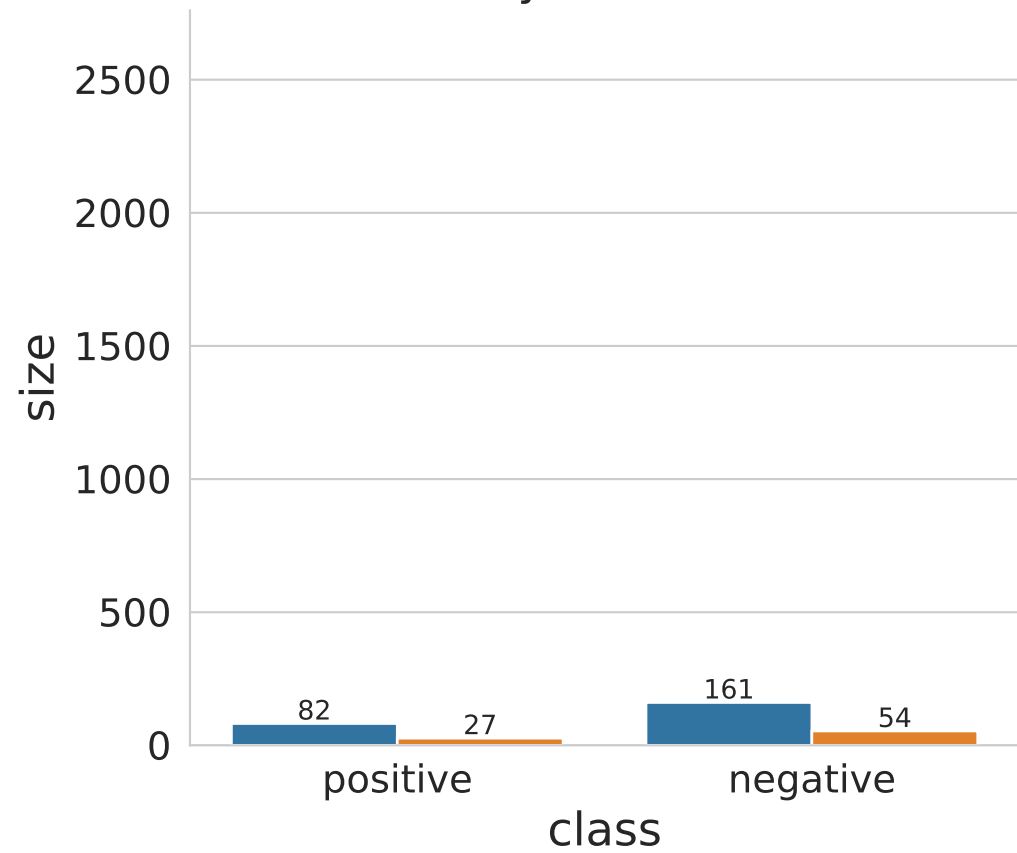

task = key count extraction

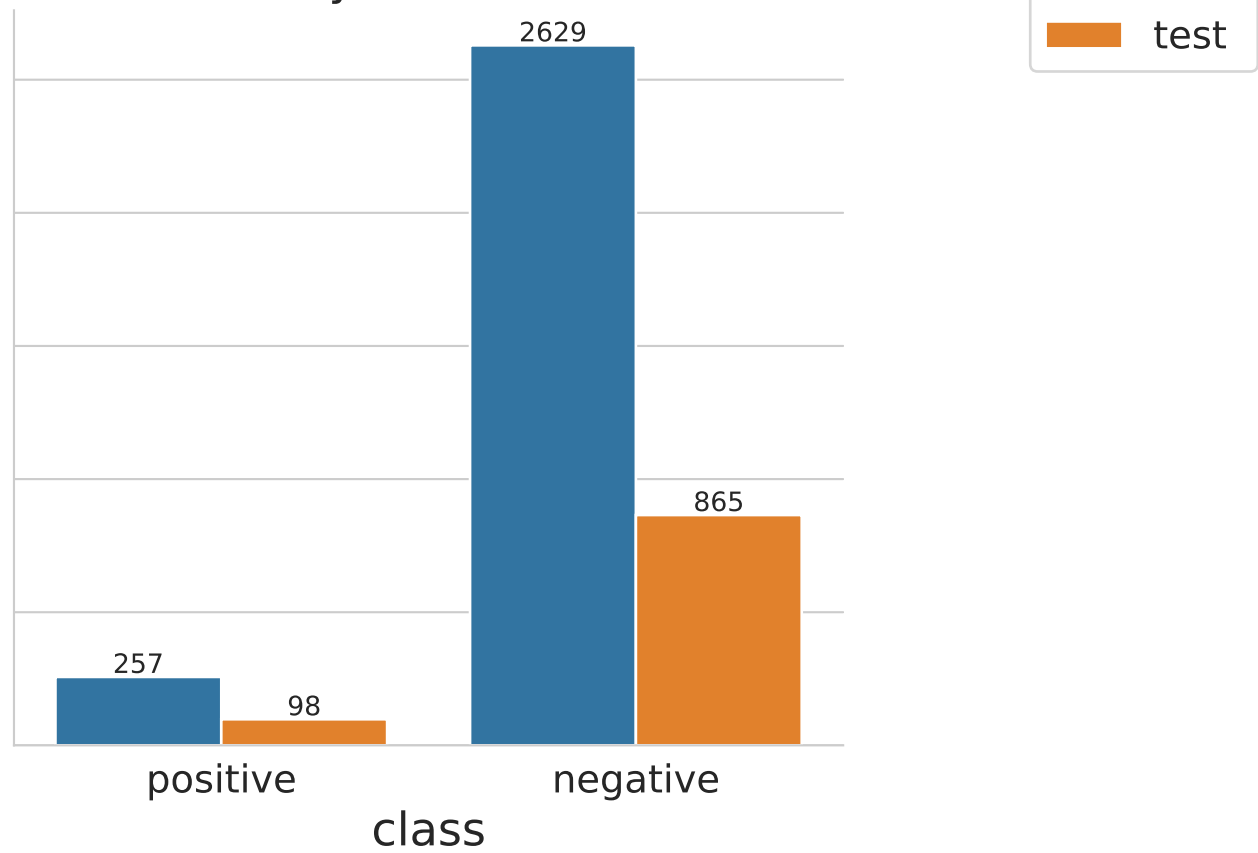

Supplement: S1 Fig — The number of articles used for each class (positive/negative, i.e. key/not key) for the partions of the dataset (train, test) are shown for each task. (PDF) [file pcbi.1008277.s004.pdf]

task = relevance scoring

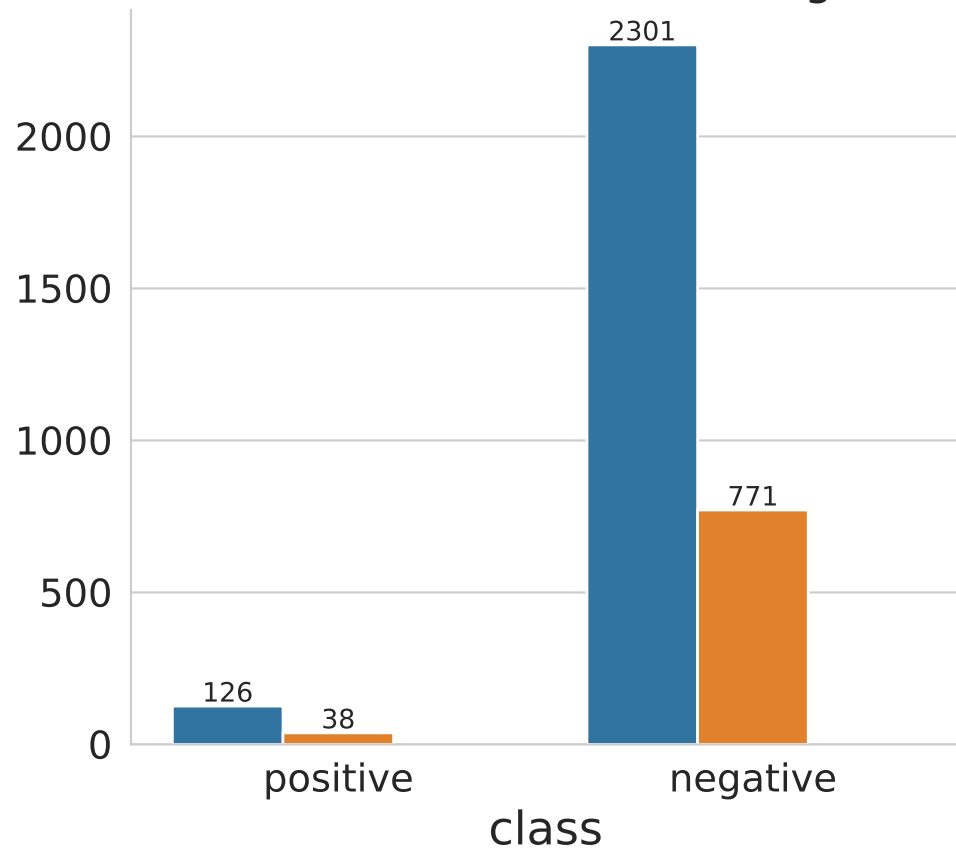

task = relevance scoring CNN

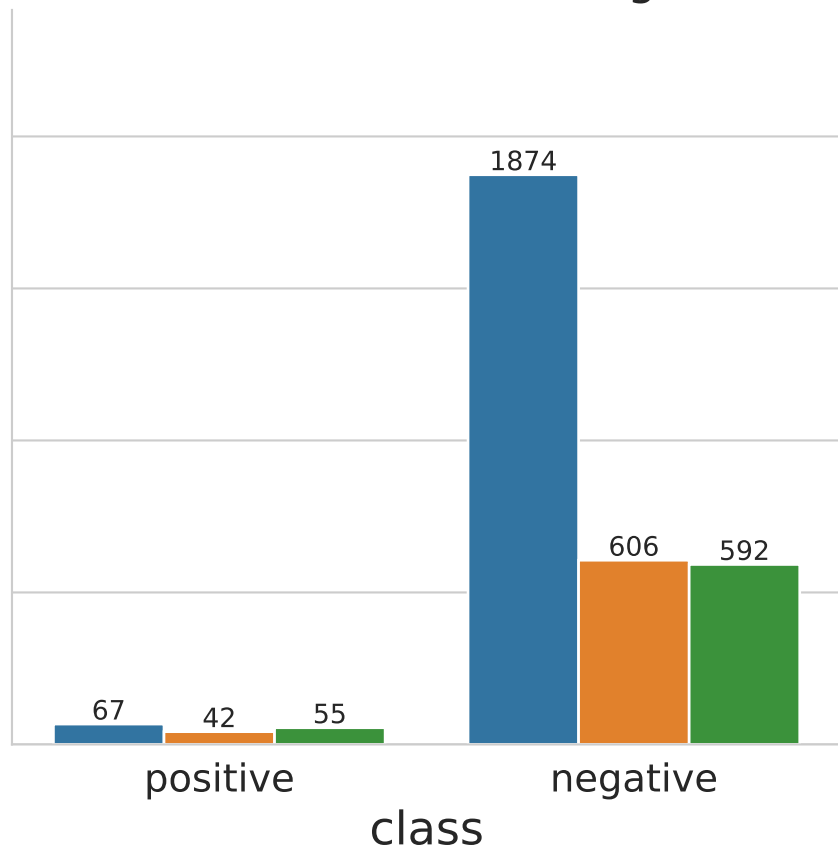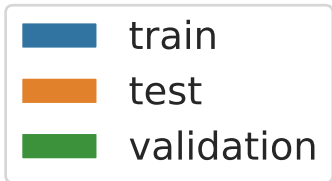

Supplement: S2 Fig — The number of articles used for each class (positive/negative, i.e. relevant/irrelevant) for the partions of the dataset (train, test, and for CNN validation) are shown for each task. (PDF) [file pcbi.1008277.s005.pdf]

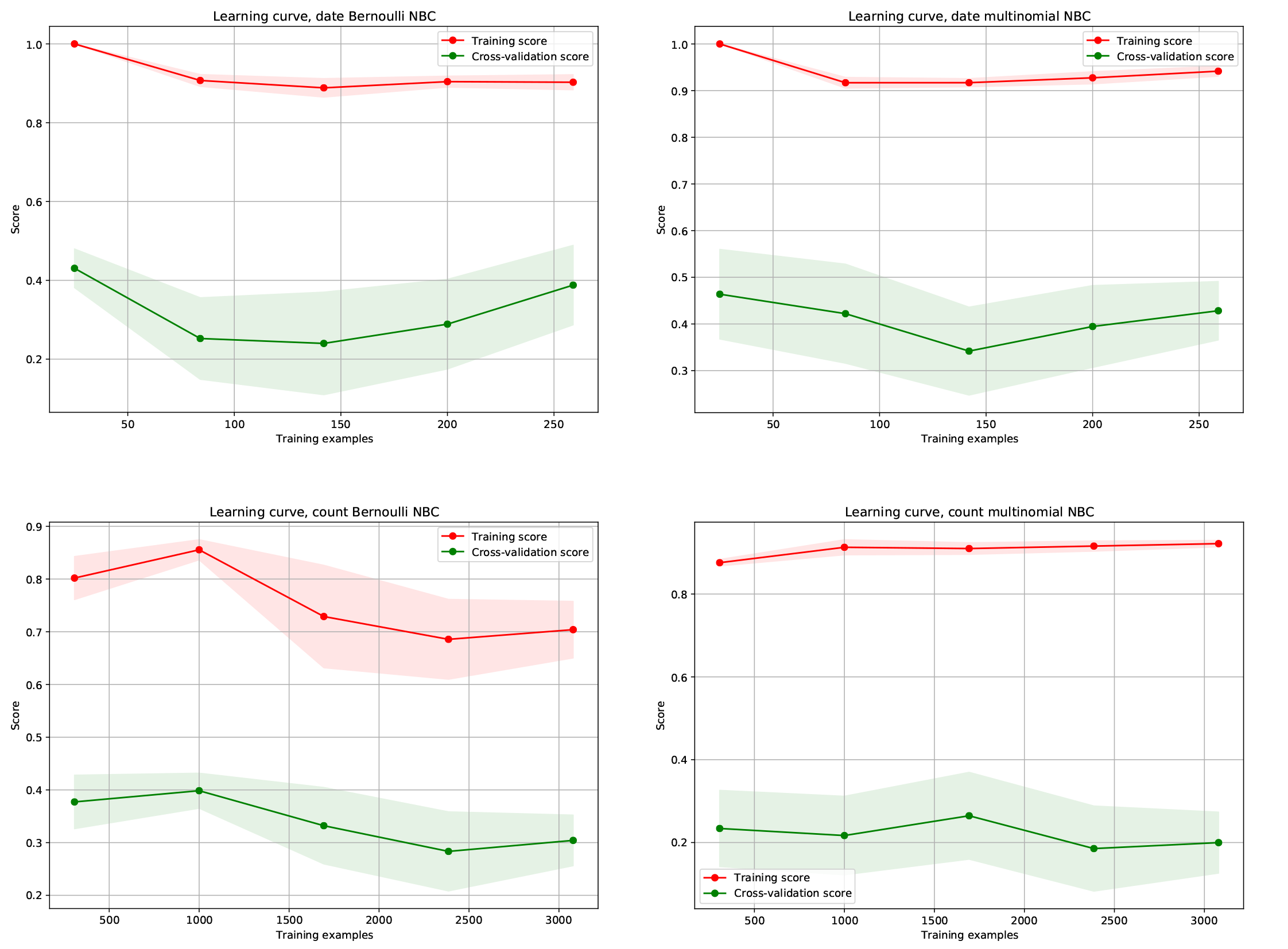

Supplement: S3 Fig — Dependency of key (date and count) classifcation performance on training data size as measured using 5-fold cross validation for the multinomial and Bernoulli naive Bayes classifiers. The performance is measured by the IBA score. The points show mean scores, the shaded regions show the mean plus and minus one standard deviation on the cross validation folds. (PNG) [file pcbi.1008277.s006.png]

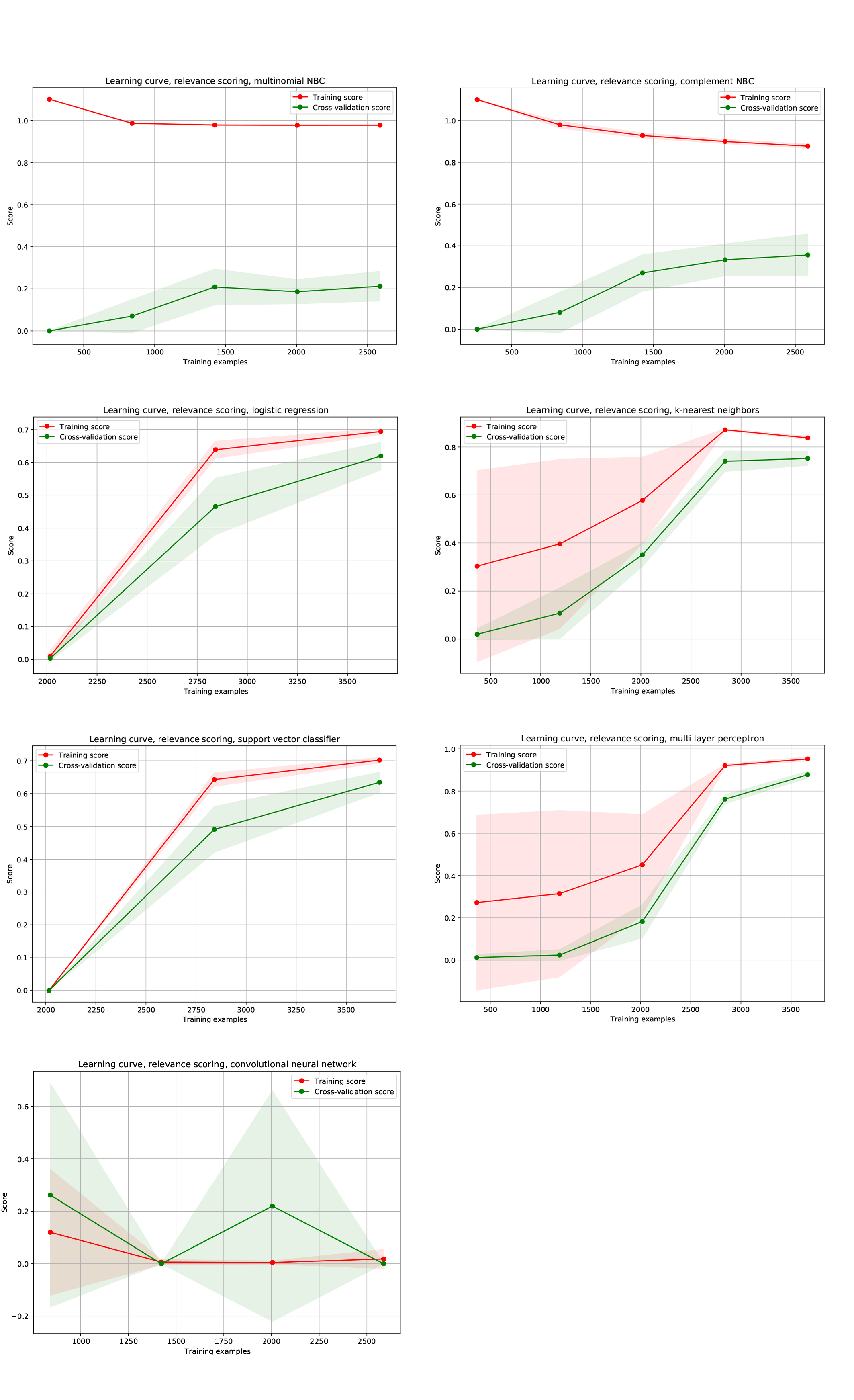

Supplement: S4 Fig — Dependency of relevance classifcation performance on training data size as measured using 5-fold cross validation for different classifiers. The performance is measured by the IBA score. The points show mean scores, the shaded regions show the mean plus and minus one standard deviation on the cross validation folds. (PNG) [file pcbi.1008277.s007.png]

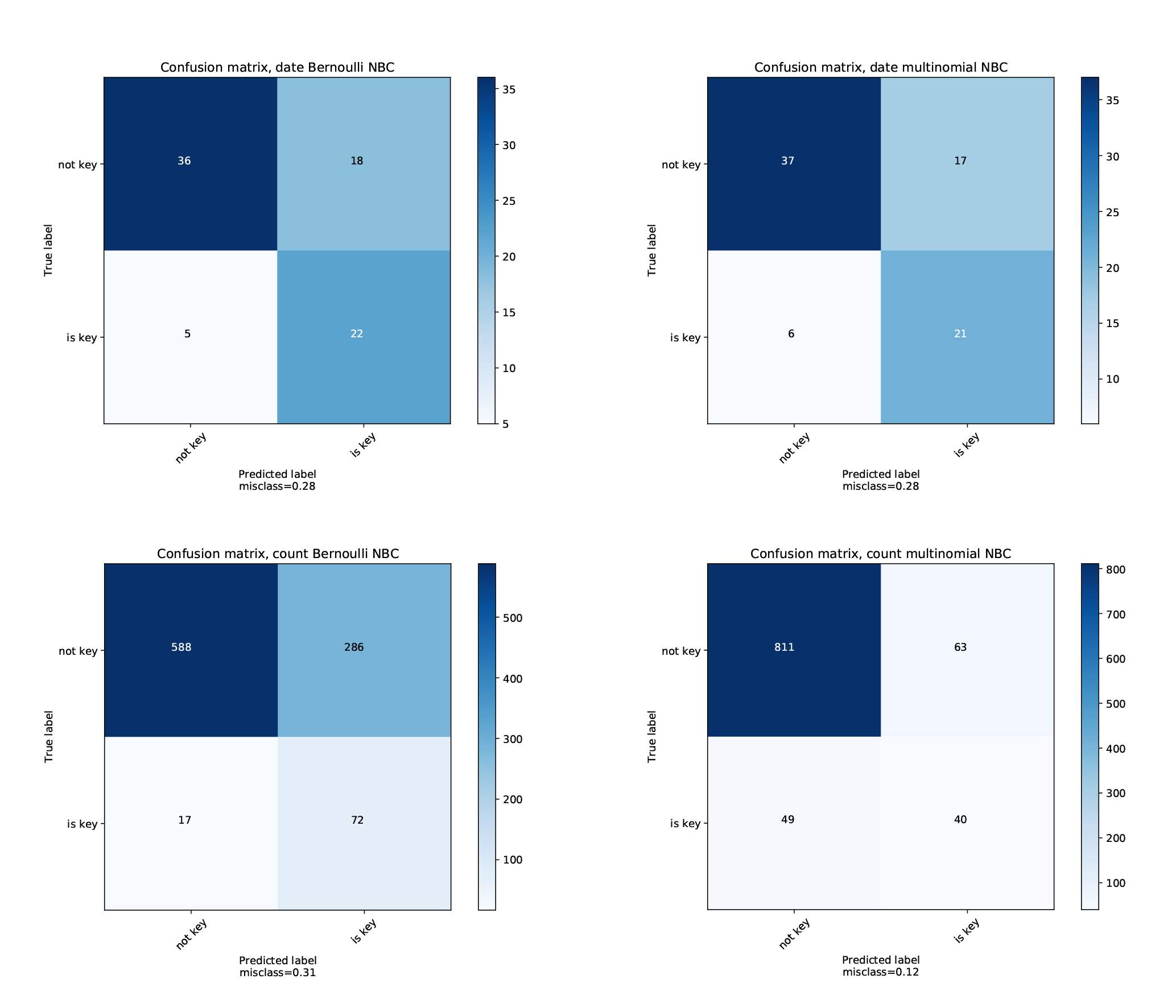

Supplement: S5 Fig — The plot shows the true and predicted labels of the test test in the key entitiy extraction task. The plots are stratified by algorithm (multinomial and Bernoulli naive Bayes classifier (NBC)) and task (key count and date extraction). Furthermore, the proportion of missclassified is shown below. (PNG) [file pcbi.1008277.s008.png]

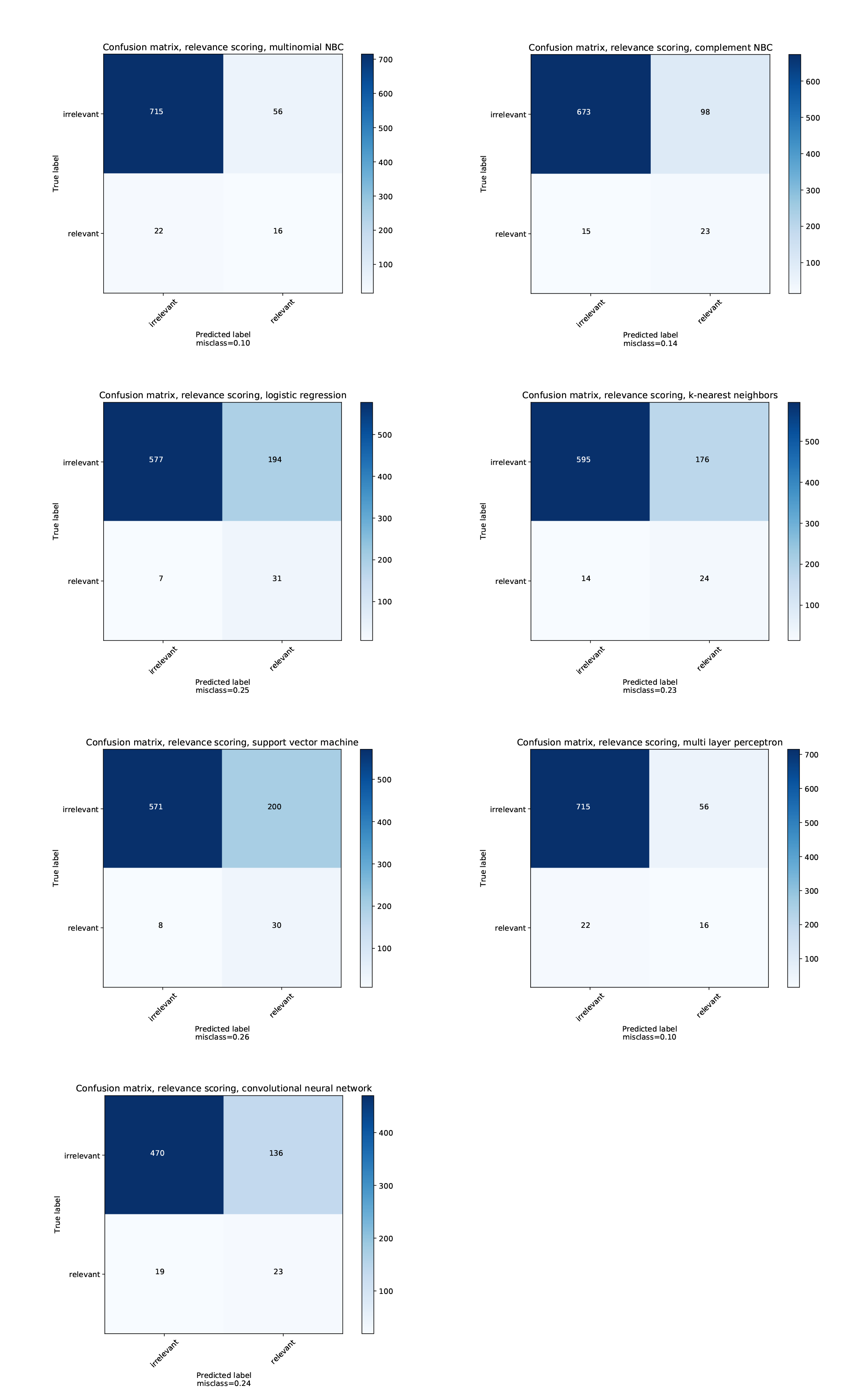

Supplement: S6 Fig — The plot shows the true and predicted labels of the relevance scoring task. The plots are stratified by algorithm. Furthermore, the proportion of missclassified is shown below. (PNG) [file pcbi.1008277.s009.png]

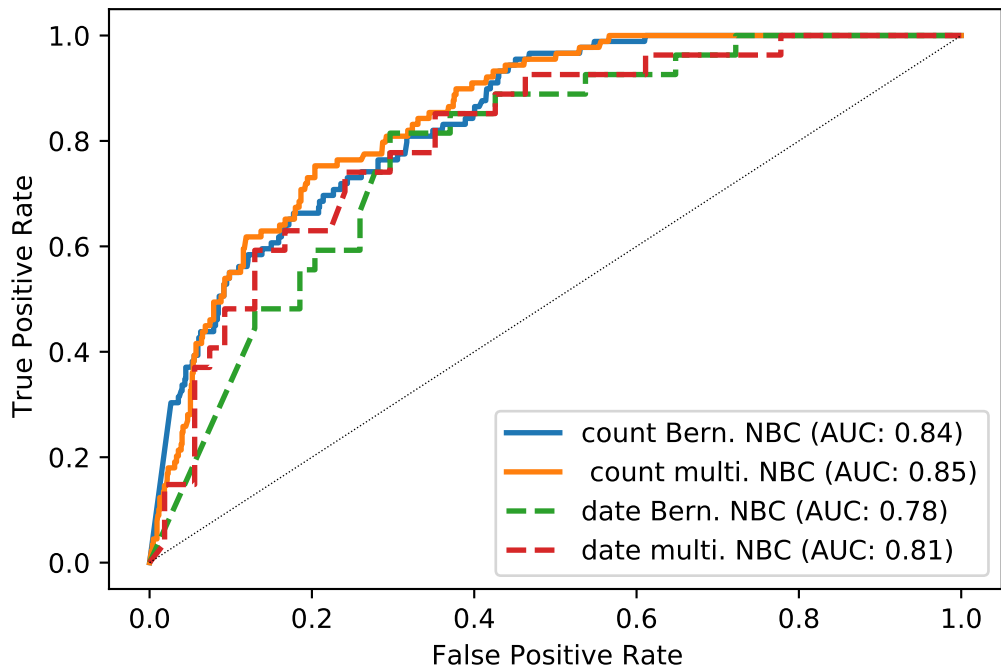

Supplement: S7 Fig — The plot shows the true positive rate against the false positive rate stratified by algorithm (multinomial and Bernoulli naive Bayes classifier (NBC)) and task (key count and date extraction) and the area under the curve (AUC). The black, dotted middle shows the expected curve for random classifcation. (PDF) [file pcbi.1008277.s010.pdf]

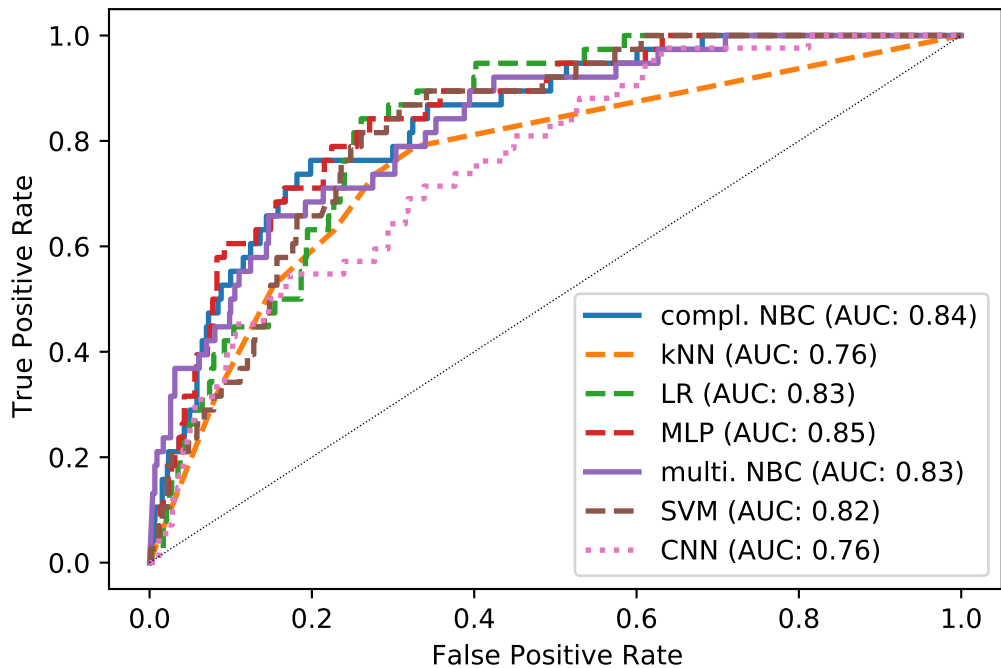

Supplement: S8 Fig — The plot shows the true positive rate against the false positive rate stratified by algorithm (complement naive Bayes classifier (compl. NBC), k-nearest neighbors (kNN), logistic regression (LR), multi layer perceptron (MLP), multi. NBC (multinomial naive Bayes classifier), support vecotor machine (SVM), and convolution neural network (CNN)) and the area under the curve (AUC). The black, dotted middle line shows the expected curve for random classifcation. (PDF) [file pcbi.1008277.s011.pdf]
