# Supplementary material for: EventEpi—A natural language processing framework for event-based surveillance
Source: PLoS Comput Biol. 2020 Nov 20;16(11):e1008277. doi: 10.1371/journal.pcbi.1008277 (PMC7717563; doi:10.1371/journal.pcbi.1008277)
Supplement: Attachment — Submitted filename: Review for PCOMPBIOL-D-19-01790.pdf [file pcbi.1008277.s012.pdf]

Review for PCOMPBIOL-D-19-01790 (EventEpi–A Natural Language Processing Framework for Event-Based Surveillance)

Overview

In the manuscript titled "EventEpi–A Natural Language Processing Framework for Event-Based Surveillance", the authors present a NLP framework that (a) builds on EpiTator to extract key date and case count from WHO DONs and ProMED posts, and (b) identifies these articles as relevant or not relevant.

Recommendation

The problem of automatic extraction of relevant information from epidemiological sources such as WHO DONs and ProMED, is both timely and important. I therefore congratulate the authors on their work, which can lead to new developments in this area. Despite the modest performance of the models presented here, I believe that the work is important. The manuscript was well-written and easy to follow. However, the readability of the manuscript can be enhanced if some details are moved to a supplementary text to make the message of the paper more crisp.
I recommend that the manuscript be **published with major revisions**.

Major Recommendations

1. Although the results from the model are modest at best, the training data set produced by the authors in the course of this work can itself be a valuable scientific product. If the data from the incident database are not confidential, I would urge the authors to consider releasing it with the manuscript. Similarly, the word and document embeddings trained on epidemiological articles if released, would allow the scientific community to build on the authors' work.
2. lines 175-176. The authors state that "the multinomial NBC often is preferred for longer text documents" and cite ref 22 to support this. The abstract of ref 22 states that "...multinomial performs even better at larger vocabulary sizes - providing an average of 27% reduction in error over the multivariate Bernoulli model **at any vocabulary size**." (emphasis mine). Ref 22 thus does not quite support the assertion.
3. lines 217-227. As the imbalance in datasets is a crucial point, I would suggest some sensitivity analysis around the method selected for up-sampling.
4. Tables 2, 3 and 4. The authors should consider including in the main text or in a supplementary note, the ROC curve for these classifiers.

Other Recommendations

1. page 4, line 7: I was unable to understand what this sentence means, consider rephrasing.
2. line 52. I could not access the GRITS webpage, consider referencing their github repository, rather than their webpage.
3. line 57. "GRITS does not filter news", it is not clear what the authors want to say by "filter news", consider expanding this point.
4. lines 68 - 70. From what I have understood, the pre-processing of the IDB is a step that has to be taken only once, (if this software is intended to be used in a continuous manner). Cam the authors please clarify?
5. lines 86: The authors have given the details of key entity filtering in a later section. However, to help the reader better understand the current section, they could perhaps add a sentence about what the filtering entailed.
6. lines 90-92. The flow here is a bit jarring, as the authors have already talked about filtering entities in the previous paragraph. The argument might flow more fluently if the authors move this sentence up, and then add a sentence about filtering, as suggested above.
7. Section "Preprocessing of the IDB", lines 96-100, lines 114-129. This section is too detailed. Details about pre-processing should, in my opinion, be presented in supplementary text.
8. line 122. It would be interesting to discuss these edge cases in a supplementary note. For instance, I was curious to know the number of cases where multiple countries were associated with a case count. Another minor point is that the authors refer to "spreading a row". I think this terminology is specific to python libraries, or R packages (such as tidyr) and is not commonly used.
9. line 128. the authors can consider briefly discussing how they selected a threshold of 40% for correcting a word. How did changing this threshold affect the number of words that had to be corrected?
10. line 154. The authors have already mentioned NLTK work tokenizer (in line 139).
11. line 155-157. As the authors rightly point out, lower casing words and removing stop words and punctuations are standard steps in NLP methods. These need not be presented here and can be moved to a supplementary note if necessary.
12. lines 159-161. The sentence beginning "We decided that they key..." is quite convoluted and I could not understand what the authors intended to convey.
13. lines 181-195. This section is again too detailed and compressing it would enhance the readability of the manuscript. In the main text, I would like to see for example, how many sources INIG has and of these, how many were used, briefly alluding to the reasons. A list of sources can be included in a supplementary note, if this is feasible.
14. lines 229-230 it would help the reader if the authors could briefly explain what IBA is measuring - an intuitive explanation would be highly appreciated.
15. Line 231. alpha is a dominance index. What is a dominance index?
16. line 235. "weighted average of the IBAs" - weighted by what?
17. lines 238-255. To improve the readability of the manuscript, the authors can present this information in a tabular form stratified by for instance, whether a method uses

document-embedding, or word embeddings. The crucial parameters for each method can be included in the table as well.

18. line 266. This information is repeated multiple times. State this once, perhaps in the supplementary text.
19. Lines 288. Use full forms of TPR and TNR the first time they are used.
20. Lines 302. The authors could consider performing this analysis on a subset of the data, and then the full dataset. This would hopefully illustrate that having more data improves the model performance (which is indeed modest, as the authors rightly acknowledge).
21. Tables 2 and 3 - the authors have not introduced F1 score. They should do so and provided a reference if possible.
22. Lines 318. Details of this "separate process of sampling" should be included in a supplementary note.
23. Lines 339. Can the authors talk a little bit about the computation time needed for training and running the various models?
24. Line 350. This contradicts the assertion made in the abstract. I believe that the authors are more realistic in their claims in the discussion than they are in the abstract.
25. The authors should include a reference to WHO DONs and ProMED.
