# Supplementary material for: EventEpi—A natural language processing framework for event-based surveillance
Source: PLoS Comput Biol. 2020 Nov 20;16(11):e1008277. doi: 10.1371/journal.pcbi.1008277 (PMC7717563; doi:10.1371/journal.pcbi.1008277)
Supplement: Attachment — Submitted filename: response.pdf [file pcbi.1008277.s013.pdf]

Dear Editors,

we are thankful for the reviewers' time and valuable suggestions. By taking their advises and comments into account, we were able to make some major revisions which hopefully improved the manuscript's quality and readability.

A large and rewarding change was to move rather detailed, technical content into a supplement. The latter now includes implementation details such as (standard) data preprocessing steps and the hyperparameters of the models. Furthermore, additional figures illustrate the classifiers' performance and bolster claims made in the manuscript. Finally, we incorporated suggestions around the understandability: We hope the manuscript has gotten closer to being interesting to read for both public health and machine learning researchers.

While going through the proposed changes we also took the chance to improve the classification performances: We fixed parts of the scraping scripts that could produce artifacts and then cause unexpected behavior in the text preprocessing. We also revised the key entity extraction which lead to slightly different performances, but still very similar to the ones indicated in the previous version of the manuscript.

Besides fixing typos and clarifying some sentences, we made one change not requested by the reviewers: We added the following sentence at the end of the Conclusion, as it is in our opinion an important message of this paper and frames it in the broader perspective of developing artificial intelligence and machine learning for public health, lines 343-347:

"This work demonstrates how machine learning methods can be applied meaningfully in public health using data readily available: As experts evaluate and document events as part of their daily work, valuable labeled datasets are routinely produced. If systematically gathered and cataloged, these offer immense potential for the development of artificial intelligence in public health."

Note that we updated the author affiliations to reflect that they changed between initial submission and manuscript revision (which includes changes to the text and figures as well improving and re-applying the analysis methods).

In the following we will go through the reviewers' comments, reply to each and show how we adopted them. We indicate for each the corresponding line numbers, figures and tables of the revised manuscript.

We hope to have adopted their suggestions sensibly and are looking forward to their response.

Sincerely,

Auss Abbood, Alexander Ullrich, Rüdiger Busche, and Stéphane Ghozzi

Suggestions

Reviewer 1

Major Recommendations

- 1

  > Although the results from the model are modest at best, the training data set produced by the authors in the course of this work can itself be a valuable scientific product. If the data from the

> incident database are not confidential, I would urge the authors to consider releasing it with the manuscript. Similarly, the word and document embeddings trained on epidemiological articles if released, would allow the scientific community to build on the authors' work.

We are pleased to say that we obtained authorization to publish the parts of the incident data base (IDB) used in this manuscript. Regarding the word embeddings: The WHO has not answered our request to publish them (they have been partly trained on WHO DON texts), but given the very abstract nature of word embeddings, that are more or less a statistic of combinations of words and thus from our understanding do not contain copyrighted material, we will provide them. (Unfortunately the data protection officer of the Robert Koch Institute hasn't replied to repeated request of advice.) ProMED has authorized us to do so.

The data (IDB and word embeddings) will be submitted to PLOS Computational Biology as supplementary material.

The source code is freely accessible on GitHub under a GNU license (https://github.com/aauss/EventEpi), the data, IDB and word embeddings have been uploaded on Figshare under a CC BY 4.0 license (https://doi.org/10.6084/m9.figshare.12575978 and https://doi.org/10.6084/m9.figshare.12575966 respectively). We have modified the manuscript to indicate that:

- Last sentence of the Abstract (under Discussion): "The source code and data are publicly available under open licenses."
- Lines 54-57, in the subsection Motivation and contribution of the Introduction: "All code and data necessary to reproduce this work are freely available under open licenses: The source code can be found on GitHub under a GNU license [7], the IDB and word embeddings (see Sec. Training of the classifiers) on Figshare under a CC BY 4.0 license, [8] and [9] respectively." The citations refer to the links to the repositories.

- 2

> lines 175-176.The authors state that "the multinomial NBC often is preferred for longer text documents"and cite ref 22 to support this. The abstract of ref 22 states that "...multinomial performs even better at larger vocabulary sizes -providing an average of 27% reduction in error over the multivariate Bernoulli model at any vocabulary size."(emphasis mine). Ref 22 thus does not quite support the assertion.

We reread the reference about multinomial naive Bayes classifiers, found that the assertion the it worked better on short texts was not supported, and corrected our assertion:

- Lines 140-142: "The Bernoulli NBC might perform better on a small vocabulary, while the multinomial NBC usually performs equally well or even better on a large vocabulary [23]."

- 3

> lines 217-227. As the imbalance in datasets is a crucial point, I would suggest some sensitivity analysis around the method selected for up-sampling.4.Tables 2, 3 and 4. The authors should consider including in the main text or in a supplementary note, the ROC curve for these classifiers.

We compared the classifiers' performance with and without up-sampling:

- lines 252-254: "A comparison on classifier trained on non up-sampled data showed no better performance than sensitivity of 0.03 and IBA of 0.02 (see S2 Table)."

We added the results without up-sampling to the supplement (S5 Table). Indeed, the learning algorithms perform very poorly without up-sampling. Additionally, we provide the ROC curves for all classifiers (S10 and S11 Figure) in the supplement.

Other Recommendations

- 4

  > page 4, line 7: I was unable to understand what this sentence means, consider rephrasing.

  The general sentence on traditional reporting systems has been removed since it did not contain necessary information.

- 5

  > line 52. I could not access the GRITS webpage, consider referencing their github repository, rather than their webpage.

  The link to GRITS was replaced by a link to the GitHub repository of its frontend component (line 59).

- 6

  > line 57. "GRITS does not filter news", it is not clear what the authors want to say by "filter news", consider expanding this point.

  "GRITS does not filter news" was meant to say that it does not include the relevance filtering capabilities presented in this paper. This sentence has been rephrased:

  - Lines 64-65: "Furthermore, GRITS does not filter texts for relevance but only extracts entities from provided texts."

- 7

  > lines 68-70. From what I have understood, the pre-processing of the IDB is a step that has to be taken only once, (if this software is intended to be used in a continuous manner). Cam the authors please clarify?

  When we mentioned preprocessing of the incident data base (IDB) at the beginning of the Material and methods, we wanted to state that preprocessing of the IDB needs to happen before scraping articles. We removed the misleading "For each part [of the pipeline]" and make clear IDB preprocessing and scraping of online articles happen successively:

  - lines 74-75: "After preprocessing the IDB, texts of articles from the RKI’s main online sources have to be extracted."

- 8

> lines 86: The authors have given the details of key entity filtering in a later section. However, to help the reader better understand the current section, they could perhaps add a sentence about what the filtering entailed.

We added a sentence on key entity filtering to the paragraph of the Material and methods where it is first mentioned. The meaning of the paragraph is now easier to understand without having read the later section:

- Lines 93-95: "To find the key entities among those found by EpiTator, we explored two methods, a heuristic and classification-based approach which we refer to as key entity filtering (see Sec. Key entity filtering)."

- 9

> lines 90-92. The flow here is a bit jarring, as the authors have already talked about filtering entities in the previous paragraph. The argument might flow more fluently if the authors move this sentence up, and then add a sentence about filtering, as suggested above.

We restructured the phrase on preprocessing of the IDB before it can be compared to the output of EpiTator and how this output had to be filtered. We made it shorter to avoid redundancy, see the paragraph on lines 93-97, and in particular:

- Lines 91-93: "Before we could explore methods to find the aforementioned key entities, we applied standard cleaning and preprocessing to the IDB such that it could be fed into machine learning algorithms (see S1 Appendix)."
- Lines 95-97: "If the filtered output of EpiTator for a given article matched the respective key information of the IDB, we then knew that the filter selected the correct key entities."

- 10

> Section "Preprocessing of the IDB", lines 96-100, lines 114-129. This section is too detailed. Details about pre-processing should, in my opinion, be presented in supplementary text.

Details about preprocessing of the IDB have been moved to the supplementary text (S1 IDB preprocessing).

- 11

> line 122. It would be interesting to discuss these edge cases in a supplementary note. For instance, I was curious to know the number of cases where multiple countries were associated with a case count.

We removed the sentences on multiple entities being entered in for a given class in the IDB as these cases, contrary to what the previous version of the manuscript indicated, actually did not come up in the data we used.

This case would however be automatically handled by EventEpi: The entry would be split in as many entries as there are entities of a given class.

- 11b

> Another minor point is that the authors refer to "spreading a row". I think this terminology is specific to python libraries, or R packages (such as tidyr) and is not commonly used.

The sentences on reorganizing the shape of the IDB have been rephrased to avoid the spreading terminology. Instead we explicitly describe what has been done (now in the supplementary text):

- Lines 521-522: "To maximize the number of IDB entries that were usable by machine learning, we transformed the dataset into a tidy format [50]."

- 12

> line 128. the authors can consider briefly discussing how they selected a threshold of 40% for correcting a word. How did changing this threshold affect the number of words that had to be corrected?

For the sake of simplicity and to avoid introducing arbitrarily set parameters, we no longer use the Levenshtein distance between entries of the IDB and reference terms to match the former to the latter, and thus no longer apply a threshold for the matching. Instead we simply used hand-written rules given that only a dozen typos needed correction.

- 13

> line 154. The authors have already mentioned NLTK work tokenizer (in line 139).

We have removed the second mention of the NLTK work tokenizer. All the implementation details, if not necessary for the overall understanding of the work, are now found in the supplementary text (S1 Text preprocessing).

- 14

> line 155-157. As the authors rightly point out, lower casing words and removing stop words and punctuations are standard steps in NLP methods. These need not be presented here and can be moved to a supplementary note if necessary.

Details about standard text preprocessing, including lower casing and removing stop words, have been moved to the supplementary text (S1 Text preprocessing).

- 15

> lines 159-161. The sentence beginning "We decided that they key..."is quite convoluted and I could not understand what the authors intended to convey.

The sentence has been rephrased to be clearer (122-124). We clarified the sentence on how one entity was selected if many of the same class appeared in a text:

- Lines 126-128: "The key information for class c was set to the entity recognized in the sentence that has the highest probability of being key and contains a recognized entity of class c."

- 16

> lines 181-195. This section is again too detailed and compressing it would enhance the readability of the manuscript. In the main text, I would like to see for example, how many

> sources INIG has and of these, how many were used, briefly alluding to the reasons. A list of sources can be included in a supplementary note, if this is feasible.

The subsection on how online sources of the IDB where selected for the analysis has been moved almost completely to the supplementary text (S1 Building the dataset). We added the list of sources used by INIG to the supplementary text (S1 Building the dataset) and kept in the main text the part mentioning dataset sizes:

- Lines 148-150: "We had access to all assessments of the IDB of the year 2018 and therefore scraped all WHO DON and ProMED articles of the year 2018. This resulted in a dataset of 3236 articles, 164 of them labeled relevant and 3072 irrelevant."

- 17

> lines 229-230 it would help the reader if the authors could briefly explain what IBA is measuring -an intuitive explanation would be highly appreciated.

An explanation of the IBA score and some intuition to this metric have been added when introducing it:

- Lines 224-227: "IBA measures the trade-off between global performance and a signed index that accounts for imbalanced predictions. It favors classifiers with better true positive rates, assuming that correct predictions on the positive class are more important than true negative predictions."

- 18

> Line 231. alpha is a dominance index. What is a dominance index?

The explanation of the dominance index of IBA has been clarified:

- Line 222: "where sensitivity − specificity is called the dominance and $0 \leq \alpha \leq 1$ is a weighting factor that can be fine-tuned based on how significant the dominating class is supposed to be."

- 19

> line 235. "weighted average of the IBAs"-weighted by what?

The sentence on the weighting of the IBA and has been removed: for coherence, and because it better reflect the goal of finding relevant articles, all scores are now computed for the positive class (key or relevant).

- 20

> lines 238-255. To improve the readability of the manuscript, the authors can present this information in a tabular form stratified by for instance, whether a method uses document-embedding, or word embeddings. The crucial parameters for each method can be included in the table as well.

We have added a table to the supplement which lists the hyperparameters of the algorithms, which vectorization was used (bag-of-words, document embeddings or word embeddings) and which algorithms used up-sampling (S4 Table).

- 21

> line 266. This information is repeated multiple times. State this once, perhaps in the supplementary text.

The redundant sentence on text preprocessing has been removed from the end of the subsection Training the classifiers. All information concerning text preprocessing has been put once in supplementary text (S1 Text preprocessing, lines 542-551).

- 22

> Lines 288. Use full forms of TPR and TNR the first time they are used.

Full forms for true positives, true negatives, false positives and false negatives are now given the first time they are used:

  - Lines 211-214: "We consider a number of scores defined as functions of true positives (TP), true negatives (TN), false positives (FP) and false negatives (FN): precision = TP/(TP + FP); sensitivity = TP/(TP + FN); specificity = TN/(TN + FP) and F1 = 2 · TP/(2 · TP + FP + FN) [37]."

Note that for simplicity we no longer mention true positive *rates* but use absolute numbers of true positives (the same applies to true negatives, false positives and false negatives). This doesn't affect the formal definition of the scores.

- 23

> Lines 302. The authors could consider performing this analysis on a subset of the data, and then the full dataset. This would hopefully illustrate that having more data improves the model performance (which is indeed modest, as the authors rightly acknowledge).

Learning curves (algorithmic performance against training set size) for all models have been produced and added to the supplement. This showed that the claim that larger datasets would improve the performance holds true for most algorithms, including all but one relevance algorithms (S6 and S7 Fig.).

- 24

> Tables 2 and 3 - the authors have not introduced F1 score. They should do so and provided a reference if possible.

We now define the F1 score before showing the values it takes and added a reference:

  - Lines 213-214: "... and F1 = 2 · TP/(2 · TP + FP + FN) [37]."

- 25

> Lines 318. Details of this "separate process of sampling" should be included in a supplementary note.

The sampling performed while splitting the data in training and test sets is now mentioned in the supplementary text and details have been provided on the libraries used:

  - Lines 538-541: "The dataset was split using sklearn and numpy [51] which supported different stratification, seed, and splitting settings. We chose the least complex splitting settings which

yielded slightly different dataset sizes for the CNN (split with numpy) and the other relevance scoring algorithms (split with sklearn)."

- 26

> Lines 339. Can the authors talk a little bit about the computation time needed for training and running the various models?

Computation time and resources have been added to the supplementary text (S1 Computational time and resources).

- 27

> Line 350. This contradicts the assertion made in the abstract. I believe that the authors are more realistic in their claims in the discussion than they are in the abstract.

We modified the Methods and Findings subsection of the Abstract to better reflect the performance of key extraction:

  - "We extracted the key country and disease using a heuristic with good results. We trained a naive Bayes classifier to find the key date and confirmed-case count, using the RKI's EBS database as labels which performed modestly."

The 4th point of the subsection What did researchers do and find of the Author summary has been rephrased to better reflect the performance of key entity extraction:

  - "We were able to extract key information from epidemiological texts such as disease, outbreak country, cases counts, and the date of these counts. While disease and country could be extracted with high accuracy, date and count could still be extracted with medium accuracy with the help of machine learning models."

We also rewrote the sentence in the Conclusion explaining that we used a well-performing heuristic and modestly performing classification approach for key entity extraction:

  - Lines 311-313: "While key disease and country can satisfactorily be extracted, the performance of key date and confirmed-case count extractions needs to be improved."

- 28

> The authors should include a reference to WHO DONs and ProMED.

We now cite a scientific article describing ProMED as well as a link to the website. We did not find a similar reference for WHO DONs and provide only a link to the website. (Links are now all provided in the references.)

  - Lines 106-107: "For the learning approach, we took the texts of the articles published in 2018 from the two most relevant sources, WHO DONs [19] and ProMED [20,21] ..."

Reviewer 2

General

- 1

> Please use “Public health surveillance” instead of “Infectious disease epidemiology” or “epidemiological surveillance” in the context of this paper.

The terms "Infectious disease epidemiology" have been replaced with "Public health surveillance" throughout the manuscript.

- 2

> Avoid the use of NLP acronym, consider using the full term “natural language processing” across the text?

The "NLP" has been replaced with "natural language processing" throughout the manuscript.

- 4

> Please define each acronym at least once (for example TPR: true predictive ratio).

Each acronym is now written out before being abbreviated, see e.g.:

  - Lines 211-214: "We consider a number of scores defined as functions of true positives (TP), true negatives (TN), false positives (FP) and false negatives (FN): precision = TP/(TP + FP); sensitivity = TP/(TP + FN); specificity = TN/(TN + FP) and F1 = 2 · TP/(2 · TP + FP + FN) [37]."

Note that for simplicity we no longer mention true positive *rates* but use absolute numbers of true positives (the same applies to true negatives, false positives and false negatives). This doesn't affect the formal definition of the scores.

- 5

> Specify what you mean by “disease”. Is this limited to laboratory specific diseases such as measles, cholera or yellow fever; or it also includes syndromes such as cutaneous rash, watery diarrhoea, jaundice? This is especially important for EBS, as its purpose is mainly to detect unknown or unexpected diseases that cannot be well captured by the routine data reporting performed by healthcare facilities.

We added an additional sentence explaining that we don't focus on syndromes and why, although this is an important part of disease surveillance. (This is because the IDB which only rarely contains syndromes or symptoms and EpiTator recognizes only a few syndromes.)

  - Lines 51-53: "We did not formally define what a “disease” was but rather followed the conventions at INIG. Although considering symptoms or syndromes might lead to earlier event detection, those were rarely entered in the IDB."
  - Lines 515-517: "Rarely, the IDB contained symptoms and syndromes instead of a disease name. We discarded symptoms (“cough” and “fever”) and kept syndromes when EpiTator could recognize them."

Author summary

- 6

> What did this research do and find: 4th point (last): misleading sentence, as mentioned in the results only countries and diseases were correctly detected, not dates or counts.

The 4th point of the subsection What did researchers do and find of the Author summary has been rephrased to better reflect the performance of key entity extraction:

- "We were able to extract key information from epidemiological texts such as disease, outbreak country, cases counts, and the date of these counts. While disease and country could be extracted with high accuracy, date and count could still be extracted with medium accuracy with the help of machine learning models."

We modified the Methods and Findings subsection of the Abstract to better reflect the performance of key extraction:

- "We extracted the key country and disease using a heuristic with good results. We trained a naive Bayes classifier to find the key date and confirmed-case count, using the RKI's EBS database as labels which performed modestly."

We also rewrote the sentence in the Conclusion explaining that we used a well-performing heuristic and modestly performing classification approach for key entity extraction:

- Lines 311-313: "While key disease and country can satisfactorily be extracted, the performance of key date and confirmed-case count extractions needs to be improved."

Introduction

- 7

> Line 3 – 4: One of the most important goals of "Public health surveillance" is the "timely" detection and response to an acute public health event; the other being to monitor the health status of the population to drive health policy. In this paper, early detection and response is the topic of interest, yet public health surveillance cannot be reduced to that.

The first sentence of the Introduction on the goals of public health surveillance has been adapted to reflect that detection of events should be timely, and detection and response are only one aspect of public health surveillance:

- Lines 3-6: "One of the major goals of public health surveillance is the timely detection and subsequent containment of infectious disease outbreaks to minimize health consequences and the burden to the public health apparatus. Surveillance systems are an essential part of efficient early-warning mechanisms [1,2]."

- 8

> Lines 14-15: “traditional surveillance” relies on “routine reporting from healthcare facilities” and not from “laboratory confirmation” (most cases are not laboratory confirmed in many settings and for many diseases).

We changed the sentence on traditional surveillance to indicate that it relies on routine reporting from healthcare facilities:

- Lines 13-16: "Therefore, besides traditional surveillance that typically relies on routine reporting from healthcare facilities, secondary data such as weather, attendance monitoring at schools and workplaces, social media, and the web are also significant sources of information [2]."

- 9

> Lines 20-22: add “routine” in the sentence: “It enables public health agents […] recognition of human cases in the **routine** reporting system”.

The word "routine" has been added the the sentence on the advantage of EBS compared to IBS:

- Lines 19-21: "It enables public health agents to detect and report events before the recognition of human cases in the routine reporting system of the public health system [2]."

- 10

> Line 29: why the use of the word “precision” instead of ”specificity”?

The term "precision" was wrongly used in one sentence, where we should have written "specificity". The sentence has been changed accordingly:

- Lines 26-28: "This task is particularly difficult because it is important that the filter does not miss any important events (sensitivity) while being confident what to exclude (specificity)."

- 11

> Lines 45-46: why only “confirmed-case count”? And not counts of suspect cases, for example?

Confirmed case count was the quantity that INIG (the EBS unit) was predominantly interested in. While other quantities, such as suspected case count, were part of the IBS, they were not mandatory and thus very incomplete, in contrast to the confirmed case counts, date, country and disease. We changed the sentence mentioning confirmed case counts for the first time accordingly:

- Lines 44-47: "automatically extracts key entities (disease, country, confirmed-case count, and date of the case count, which are the mandatory entries of the IDB and thus are complete) from an epidemiological article and puts them in a database, making tedious data entry unnecessary;"

Figure 1

- 12

> Don't use the light "pink-orange" background for boxes as it is confusing with the orange part of the figure describing the relevance scoring.

The background of the boxes of the pipeline diagram have changed to a more neutral color (light grey) to avoid confusion with colors used in other parts of the diagram (Fig. 1).

- 13

> Define briefly "word2vec", "EpiTator", "SVM", "kNN", "LR" either in the figure or in its description.

Explanations of the abbreviations used in the diagram have been added to the figure description (Fig. 1).

- 14

> To facilitate understanding, maybe consider splitting the "supervised learning" row in two: the classification phase (relevance scoring), and the identification of the appropriate data (key information extraction). And use the terms "relevance scoring" and "key information extraction" in the figure.

As suggested, the pipeline diagram now has boxes split in two distinct classification steps to clearly show that key entity extraction and relevance scoring happen independently. The terms "key entity extraction" and "relevance scoring" are now used in the diagram instead of the more generic "classification". (Fig. 1)

Material and methods

- 15

> - Add a section to detail how you assessed the performance of the key information extraction and of the classifiers, this would encompass among other lines 85-89, 267-273 in the methods, and some paragraphs from the results, for example lines 288 to 295.
> - In the above proposed section, please add a quick description of all indicators used to assess each extraction and classification method (i.e. indicators described in the results and in tables 2 to 4).

We added an extra section named "Evaluation" (starting line 199) which contains all the evaluation metrics and considerations used in this work, at the same time for both key entity extraction and relevance scoring. Information regarding evaluation has been removed from other sections, thus avoiding repetitions and shortening the main text.

- 17

> For epidemiologists the term “sensitivity” will be clearer than “recall”.

The term "recall" has been replaced with "sensitivity" throughout the manuscript.

- 18

> Lines 79-80: if correct, add “in each class” in the following sentence: “However INIG, as other […] in the keys entities *in each class*”.

The terms "each class" have been added to sentence introducing the concept of key entity:

- Lines 85-86: "However INIG, as other EBS groups, is mostly interested in the key entities of each class."

- 19

> Lines 120-121: you mention the problem of events/incidents involving several countries but you don’t specify how you solved it (for example to have an accurate number of cases for each country). Please specify how many events/incidents it concerned and how you solved this problem. Why didn’t you remove these events for training the algorithm?

We removed the sentences on multiple entities being entered in for a given class in the IDB as these cases, contrary to what the previous version of the manuscript indicated, actually did not come up in the data we used.

This case would however be automatically handled by EventEpi: The entry would be split in as many entries as there are entities of a given class.

- 20

> Table 1: please specify what a “sample” is; and what is considered “positive” or “negative” samples.

An explanation has been added to the legend.

- 31

> Lines 159-161: if I understood well, the key information for a single class is the recognized entity from the sentence with the highest probability. How did you deal with sentences having more than one recognized entity for a single class?

We rephrased the statement about how we ensure that a sentence is always extracted using the most probable positive classification:

  - Lines 126-130: "The key information for class c was set to the entity recognized in the sentence that has the highest probability of being key and contains a recognized entity of class c. This method ensures that some entity is still chosen even if no sentence is being classified as key, i.e., if all sentences in a text have less than 50% probability of being key."

Regarding the edge case on how to deal with more than one entity for a single class within a single sentence: Unfortunately, EventEpi does not handle this case specifically right now. We did not encounter this case during our work, however this is a good point and requires attention. We hope that EventEpi being open-source we will work together with the community to find solutions to these and other edge cases should they arise for other users.

- 32

> Please make clear the number of articles used in your samples:
>
> - As I understand, you had 3232 articles, 160 relevant (included in the IDB) and 3072 irrelevant.
> - And then you tested your classifiers with 20% of your sample, please provide figures of relevant and irrelevant articles used to test your classifiers.
> - As I understand, you had two classes: relevant or irrelevant article. If I am correct, please make it clear, including in the sections related to the test of the classifiers.

The term "support" is now explained in the legend of the results table and states how many samples were used in the test set for each algorithm. Furthermore, we added figures to show the different dataset sizes and class compositions (S4 and S5 Fig.).

Results

- 33

> When you say all countries and diseases (except one) were correctly recognized, please provide the figures; i.e. for how many articles presents in the IDB country and disease were correctly extracted. Same for dates and counts.

We went over the code to retrieve the exact numbers. We now provide the numbers and must admit to have made a mistake during the performance evaluation. The results for the key countries and diseases recognition are now slightly worse, but still quite good:

- Lines 234-237: "The most frequent approach worked well for detecting the key country (85% correctly classified) and disease (88% correctly classified). Note that EpiTator systematically failed to detect Nipah virus infection and anthrax."

- 34

> Lines 299-302: not clear what the results related to EpiTator and the ones related to the most frequent approach are.

We reformulated the last paragraph of the Performance of key date and count extraction subsection of the Results to make clear that we are referring to classification of EpiTator output, not the most frequent approach:

- Lines 245-246: "Thus, without offering perfect results, applying classification to the output of EpiTator enables key entity extraction for date and confirmed-case count."

- 35

> Please also provide figures for table 4. For example, for each classification modality, how many documents were classified as relevant and not relevant, how many were truly relevant and how many truly not relevant?

We added confusion matrices to the supplement, with absolute numbers of classified texts (true positives, true negatives, false positives, false negatives) for all algorithms (S8 and S9 Fig.).

- 36

> Similarly, it would be good to know if other incidents not logged in the IDB but still of interest were identified using the automatized screening approach, i.e. documents identified as "relevant" by the classifier, that were not in the IDB, but that should have been after a review by a public health expert.

Indeed, it would be very interesting to review all articles classified by EventEpi as positives but not as such by the public health experts at INIG: Maybe they missed something, maybe this are articles similar to others already put in (and thus relevant but not more informative)? This will be part of a future evaluation of EventEpi through users.

Unfortunately, the IDB we used is now somewhat old (dating 2018). The selecting rules and the team have changed which makes it difficult to tell whether EventEpi would have found missing **relevant** articles then. (It is in general very difficult in retrospect to gauge the relevance of an article, since from a public health agent point of view it depends on the context, e.g. whether the event described is new.).

Moreover, our colleagues at INIG are now fully engaged in the COVID-19 response, so that a user evaluation will have to be postponed of at least a few months. Finally, we believe this might be out of scope of this work which intends to show a proof of concept. Rather, it will be part of a second version where we will work on implementation details, user experience, as well as maximizing algorithmic performance.

Conclusion

- 37

> The most important added value of the tool would be to pre-screen large amounts of data to identify a sample that would be then manually screened by public health experts.

The insight that the most important added value of the tool would be to pre-screen large amounts of data has been added to the conclusion:

- Lines 308-310: "Even with limited datasets, EBS can be supported by automatic processes, such as pre-screening large amounts of news articles to forward a condensed batch of articles for manual review."
