# Supplementary material for: EventEpi—A natural language processing framework for event-based surveillance
Source: PLoS Comput Biol. 2020 Nov 20;16(11):e1008277. doi: 10.1371/journal.pcbi.1008277 (PMC7717563; doi:10.1371/journal.pcbi.1008277)
Supplement: Attachment — Submitted filename: response_2.pdf [file pcbi.1008277.s014.pdf]

Dear Editors,

we thank you for taking the time to read through our revised paper and we are glad to see that we incorporated your suggestions to your satisfaction.

In the following you will find our corrections to your newest remarks in a structured form and with references. Furthermore, we made small changes to the manuscript ourselves, which we reference as well and hope that you agree with them. We now also provide all data to directly produce plots and tables without having to rerun our analyses.

Sincerely,

Auss Abbood, Alexander Ullrich, Rüdiger Busche, and Stéphane Ghozzi

Applied changes

Suggestions made by the reviewers

- Line 40: typo, "spent" instead of "spend".
    - The new corrected line is now "The existence of SOPs and the amount of time spent with manual information extraction and especially data entry lead to the idea to automate parts of the process.", line 40.
- line 554: typo "classification" instead of "classifcation"
    - The new corrected line is now " All the classification algorithms took less then one minute to train except for the complement and multinomial NBC for the relevance scoring task which took around five minutes.", line 556-558
- Have all data underlying the figures and results presented in the manuscript been provided? Large-scale datasets should be made available via a public repository as described in the PLOS Computational Biology data availability policy, and numerical data that underlies graphs or summary statistics should be provided in spreadsheet form as supporting information. Reviewer #1: None Reviewer #2: None
    - The source code is freely accessible on GitHub under a GNU license (https://github.com/aauss/EventEpi), the data (IDB and word embeddings) have been uploaded on Figshare under a CC BY 4.0 license (https://doi.org/10.6084/m9.figshare.12575978 and https://doi.org/10.6084/m9.figshare.12575966 respectively). We have now *uploaded all CSVs to recreate the confusion matrices, learning curves, and other figures used in the manuscript* with the re-submission and to (https://github.com/aauss/EventEpi/tree/simple/data/data_for_plots). We would be happy to provide any further material required.

Changes introduced by us

Author Summary (What do these findings mean?)

- We changed the word *can* to *could* in the following phrase, "Although parts of the information extraction function robustly and the relevance scoring could already save public health agent’s time, methods to explain deep and machine learning models showed that the learned patterns are

sometimes implausible.". We thought that *could* might be more precise here since we did not extensively test the amount of saved time using *EventEpi*.

Introduction

- We added the following citation to further support the claim of the sentence "Especially on the web, the topicality and quantity of data can be useful to detect even rumors of suspected outbreaks" in line 23: Kaiser, Coulombier, Baldari, Morgan and Paquet (2006) "What is epidemic intelligence, and how is it being improved in Europe?", Euro Surveillance, https://doi.org/10.2807/esw.11.05.02892-en

Learning curves

- We have clarified the descriptions of the learning curves:
    - The legend of Fig. S3 (indicated as Fig. 6 in the PDF re-submitted) now reads: "Dependency of *key* (date and count) classifcation performance on training data size as measured using 5-fold cross validation for the multinomial and Bernoulli naive Bayes classifiers. The performance is measured by the IBA score. The points show mean scores, the shaded regions show the mean plus and minus one standard deviation on the cross validation folds."
    - The legend of Fig. S4 (indicated as Fig. 7 in the PDF re-submitted) now reads: "Dependency of *relevance* classifcation performance on training data size as measured using 5-fold cross validation for different classifiers. The performance is measured by the IBA score. The points show mean scores, the shaded regions show the mean plus and minus one standard deviation on the cross validation folds."
- We rerun the training of the algorithms to save the underlying data for the plots and figures used in the manuscript. Since we were not able to make the CNN runs deterministic, the learning curve changed a little bit in the new run. However this doesn't affect the claims made in the manuscript. We updated the learning curve of the CNN in the relevance scoring part (Fig. 7, appendix).
